# Serum parathyroid hormone trajectory during the first year of hemodialysis: a roadmap to severe hyperparathyroidism

**DOI:** 10.1590/2175-8239-JBN-2024-0182en

**Published:** 2025-06-13

**Authors:** Eduardo J. Duque, Maria Eugenia F. Canziani, Ana Beatriz L. Barra, Maria A. Dalboni, Jorge P. Strogoff-de-Matos, Rosilene M. Elias, Rosa M. A. Moysés

**Affiliations:** 1 Universidade de São Paulo, Faculdade de Medicina, Hospital das Clínicas, Laboratório de Fisiopatologia Renal, Departamento de Nefrologia, São Paulo, SP, Brazil.; 2 Universidade Federal de São Paulo, São Paulo, SP, Brazil.; 3 Universidade Federal Fluminense, Rio de Janeiro, RJ, Brazil.; 4 Universidade Nove de Julho, São Paulo, SP, Brazil.

**Keywords:** Parathyroid Hormone, Dialysis, Hyperparathyroidism, Secondary, Chronic Kidney Disease-Mineral, Bone Disorder

## Abstract

**Background:**

Data on parathyroid hormone (PTH) levels at hemodialysis (HD) initiation and during the first year of therapy are still scarce. We hypothesized that high baseline PTH levels contribute to more severe hyperparathyroidism during this period.

**Methods:**

Incident HD patients (n = 1,973) were divided into 3 groups according to PTH values (<150, 150–600, and > 600 pg/mL).

**Results:**

PTH levels at baseline and at 1 year were 273 (133–508) and 255 (128–471) pg/mL, respectively (p = 0.291). PTH < 150, 150–600 and >600 pg/mL were found in 28.1, 53.5 and 18.4%, respectively, at baseline and 30.7, 52.5 and 16.8% after 1 year (p = 0.015). Younger age, absence of diabetes, high baseline alkaline phosphatase and PTH were independent risk factors for PTH > 600 pg/mL after 1 year of HD.

**Conclusion:**

High PTH at the beginning and after 1 year of HD indicate poor conservative management before and during dialysis, and put patients at risk of requiring parathyroidectomy later.

## Introduction

Chronic kidney disease-mineral and bone disorder (CKD-MBD) is an extensive systemic disorder manifested in uremic patients. This syndrome comprises vitamin D deficiency, vascular calcification, abnormalities in bone turnover, altered metabolism of calcium and phosphate, and increased levels of fibroblast growth factor-23 (FGF-23) and parathyroid hormone (PTH), known as secondary hyperparathyroidism (SHPT).

SHPT damages several systems^1^, including bone quality, which contributes to increased fracture risk in CKD patients. SHPT results in hyperphosphatemia, increased skeletal calcification, anemia, and worse quality of life. Furthermore, it has been denoted that PTH >600 pg/mL leads to a 21% increase in all-cause mortality^2^. Studies show that patients with moderate or severe SHPT submitted to parathyroidectomy (PTX) have increased survival, improvement in symptoms such as bone pain and incidence of fractures^3^, and improvement of quality of life and functionality^4,5^.

In Brazil, the high number of patients on dialysis waiting for PTX suggests that controlling PTH is still challenging. High PTH levels in incident patients on HD are associated with more difficult control of SHPT in the long term^6^, although Brazilian data are still lacking. We hypothesized that elevated PTH levels in incident patients might contribute to the severity of SHPT during the first year of HD.

## Methods

This was a longitudinal, observational, retrospective cohort study of adult patients with end-stage renal disease on HD followed at 23 Fresenius Medical Care dialysis facilities in Brazil (Rio de Janeiro, São Paulo, Minas Gerais, Bahia, Pernambuco, and Federal District). We evaluated 1,973 of 4,317 adult individuals who started HD between February 2012 and June 2017^7^ and completed 1 year of therapy. During this period, there were 661 deaths (17.4%). We also excluded from the analysis patients who underwent peritoneal dialysis, had kidney transplantation, were transferred to other facilities, or did not have PTH measured at baseline or at 1 year of follow-up. The data were collected from electronic charts and included age, sex, race, diabetes mellitus, body mass index, paying source (Public Health System or private), place of first dialysis (outpatient facility or hospital), and levels of calcium, phosphate, albumin, urea, alkaline phosphatase and PTH.

Intact parathyroid hormone (PTH; reference range, RR 15–65 pg/mL) and serum 25-vitamin D (RR = 30–60 ng/mL for risk groups, including CKD patients) were measured by chemiluminescent immunoassay. Serum total calcium (reference range [RR] = 8.4 – 10.2 mg/dL), serum alkaline phosphatase (ALP; RR = 35–104 U/L), serum phosphate (RR = 2.3–4.5 mg/dL), and albumin (RR = 3.4 to 5.4 g/dL) were measured using colorimetric assay.

Patients were divided into 3 groups according to PTH values at baseline and after 12 months, using the cutoff points of <150, 150–600 and >600 pg/mL, which would approximately correspond to < 2 times the upper limit of the reference range (UP), 2–9 times the UP and 9 times the UP. Uncontrolled SHPT was defined as PTH >600 pg/mL.

### Statistical Analysis

Continuous variables are expressed as means and standard deviations or as medians and interquartile ranges, as appropriate. Categorical variables are expressed as absolute values and percentages, and the differences among groups were verified by the Chi-Square test. PTH values at different moments were compared by McNemar’s test.

To evaluate the effects of demographic and clinical characteristics (predictive variables) on PTH levels, univariate and multivariate logistic regressions were used. In the initial multivariate model, all predictive variables were considered. For predictive variables given in numerical and categorical form, we considered the form whose association with PTH status at twelve months was more significant or with the largest effect size. Then, variables that were not significant were excluded one by one in order of significance (backward method). For all statistical tests, a significant level of 5% was used. The analyzes were performed with SPSS 20.0 and STATA 17.

## Results

**Figure 1:**
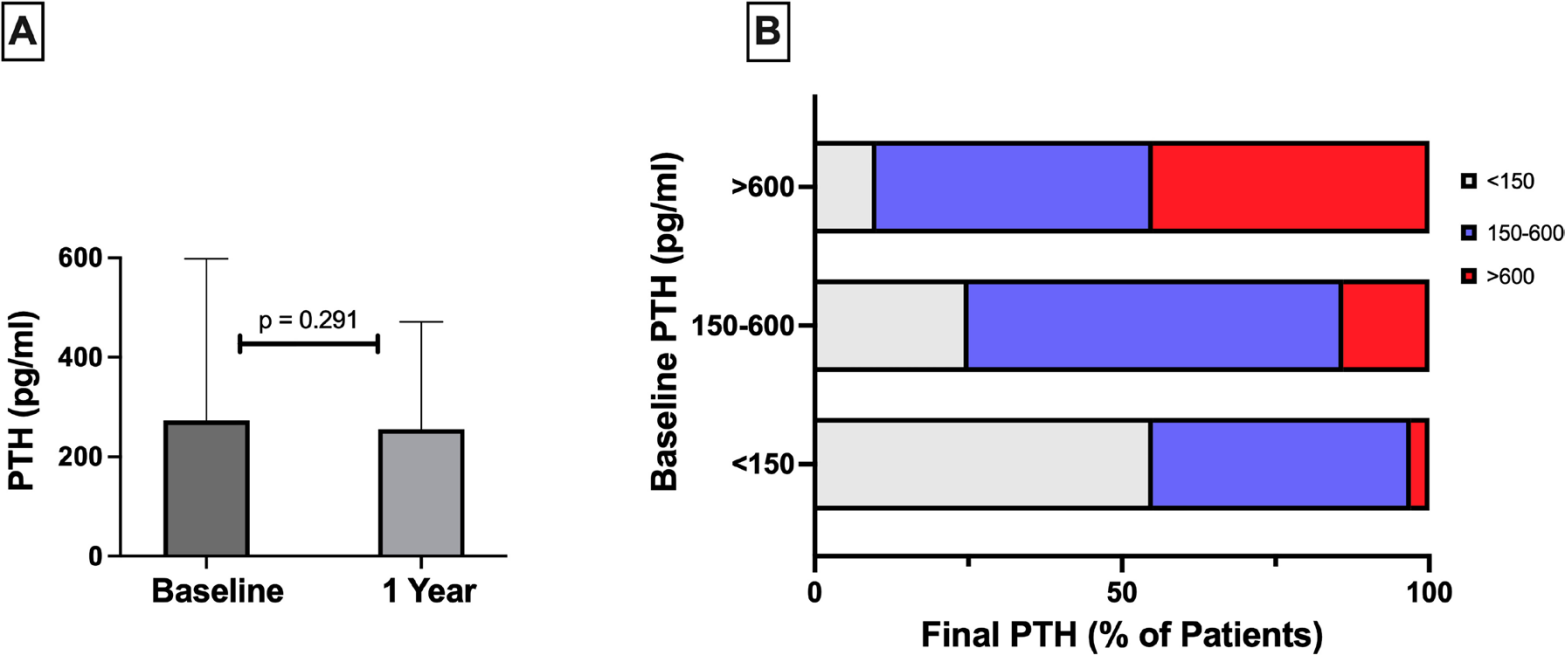
PTH levels at baseline and after 1 year of hemodialysis. A – PTH values in the study group remained stable after one year of therapy, with no significant changes in the median levels compared to baseline. B – The distribution of PTH levels at 12 months, categorized by groups of initial levels of PTH, revealed that while half of the patients with baseline PTH > 600 pg/mL experienced a decrease in their serum levels, an equivalent number of patients from other groups increased PTH levels above 600 pg/mL. Consequently, the proportion of patients with uncontrolled hyperparathyroidism remained equal to baseline.

PTH levels at baseline and at 1 year were 273 (133–508) and 255 (128–471) pg/mL, respectively (p = 0.291), as shown in Figure 1A. When starting HD, PTH levels <150, between 150–600 and >600 pg/mL were found in 28.1%, 53.5% and 18.4% of patients, respectively. After 1 year, these levels reached 30.7%, 52.5% and 16.8% of the patients (p = 0.015). Considering patients with baseline PTH >600 pg/mL, 44.9% and 10.2% reached levels of 150–600 and <150 pg/mL, respectively, and 44.9% remained with PTH >600 pg/mL after 1 year of follow-up (Figure 1B). Regarding the patients with initial PTH below 150 pg/mL, 41.6% changed to the range of PTH between 150 and 600 pg/mL, and 2.9% developed PTH > 600 pg/mL after 1 year. Among patients with initial PTH between 150 and 600 pg/mL, 24.7% and 14.5% went on to have PTH below 150 and above 600 pg/mL, respectively.

As shown in Table 1, patients with PTH >600 pg/mL at 12 months of HD were younger, and more likely to be non-white, with a lower prevalence of diabetes and private paying source. In addition, at baseline, they presented higher serum PTH, phosphate, ALP and albumin. However, at 12 months, no differences were seen between the groups regarding serum phosphate, ALP or albumin.

**Table 1:** Demographic and clinical characteristics by pth status at 12 months

	PTH – 12 months		Total	p
	≤ 600	> 600		
**Gender**				0.603
Female	652 (39.7)	137 (41.3)	789 (40)	
Male	989 (60.3)	195 (58.7)	1,184 (60)	
**Age (years)**	57.0 ± 15.4	50.4 ± 15	55.9 ± 15.5	**<0.001**
**Ethnicity**				**0.024**
Nonwhite	913 (55.6)	207 (62.3)	1,120 (56.8)	
White	728 (44.4)	125 (37.7)	853 (43.2)	
**BMI (kg/m^2^)**	24.41 ± 4.86	24.85 ± 5.65	24.49 ± 5	0.254
**Diabetes**				**<0.001**
No	916 (55.8)	245 (73.8)	1,161 (58.8)	
Yes	725 (44.2)	87 (26.2)	812 (41.2)	
**Public system**				**<0.001**
No	623 (38)	88 (26.5)	711 (36)	
Yes	1,018 (62)	244 (73.5)	1,262 (64)	
**1st HD at hospital**				0.090
No	465 (29.9)	107 (34.7)	572 (30.7)	
Yes	1,092 (70.1)	201 (65.3)	1,293 (69.3)	
**BASELINE**				
**PTH baseline (pg/mL)**				**<0.001**
<150	539 (32.8)	16 (4.8)	555 (28.1)	
150 – 600	902 (55)	153 (46.1)	1,055 (53.5)	
>600	200 (12.2)	163 (49.1)	363 (18.4)	
**Calcium (mg/dL)**	8.82 ± 1.32	8.75 ± 1.37	8.81 ± 1.32	0.819
**Phosphate (mg/dL)**	4.79 ± 1.60	5.15 ± 1.53	4.85 ± 1.59	**<0.001**
**ALP (U/L)**	107.59 ± 76.50	143.33 ± 110.04	113.61 ± 84.14	**<0.001**
**Albumin (g/dL)**	3.57 ± 0.52	3.67 ± 0.50	3.59 ± 0.52	**<0.001**
**12 MONTHS**				
**Calcium (mg/dL)**	9.02 ± 1.17	9.08 ± 1.13	9.03 ± 1.16	0.395
**Phosphate (mg/dL)**	4.91 ± 1.54	5.02 ± 1.43	4.93 ± 1.52	0.121
**ALP (U/L)**	180.58 ± 279.45	171.45 ± 266.64	179.03 ± 277.27	0.672
**Albumin (g/dL)**	3.81 ± 0.45	3.81 ± 0.43	3.81 ± 0.45	0.709

Abbreviations – PTH: parathyroid hormone; BMI: body mass index; HD: hemodialysis; ALP: alkaline phosphatase. Notes – Data are presented as mean ± SD or median (IQR), except where otherwise indicated. Bold data indicate significance.

Multivariate analysis (Table 2) confirmed that age [OR 0.981 (0.973–0.990)] and diabetes [OR 0.595 (0.445–0.795)] were negatively associated, whereas baseline ALP [OR 1.003 (1.001–1.004)] and baseline PTH > 600 pg/mL [OR 4.003 (3.055–5.325)] were positively associated with the presence of uncontrolled hyperparathyroidism at the end of the first year of therapy.

**Table 2:** Univariate and multivariate logistic regression analysis between demographic parameters/laboratory results and development of pth > 600 Pg/Ml after 1 year of dialysis

	Univariate analysis		Initial multivariate analysis			Final multivariate analysis		
	OR (CI 95%)	p	OR (CI 95%)	p		OR (CI 95%)		p
**Male (ref. female)**	0.938 (0.738−1.193)	0.603	0.955 (0.713−1.280)	0.759	–		–	
**Age (years)**	0.973 (0.966–0.981)	**<0.001**	0.981 (0.971−0.991)	**<0.001**	0.981 (0.973−0.990)		**<0.001**	
**White (ref. nonwhite)**	0.757 (0.594−0.965)	**0.025**	0.973 (0.721−1.315)	0.861	–		–	
**BMI (kg/m^2^)**	1.017 (0.994−1.041)	0.145	1.025 (0.997−1.053)	0.084	–		–	
**Diabetes**	0.449 (0.345−0.584)	**<0.001**	0.581 (0.420−0.803)	**0.001**	0.595 (0.445−0.795)		**<0.001**	
**Public Health System**	1.697 (1.304−2.208)	**<0.001**	1.131 (0.819−1.564)	0.454	–		–	
**1st HD at hospital**	0.800 (0.618−1.036)	0.090	0.912 (0.670−1.240)	0.556	–		–	
**Baseline**								
PTH pg/dL (ref. 151 a 600)		**<0.001**		**<0.001**			**<0.001**	
<150	0.175 (0.103−0.296)	**<0.001**	0.185 (0.104−0.329)	**<0.001**	0.181 (0.106−0.307)		**<0.001**	
>600	4.805 (3.673−6.285)	**<0.001**	4.037 (2.960−5.507)	**<0.001**	4.033 (3.055−5.325)		**<0.001**	
PTH (pg/dL)	1.003 (1.003−1.004)	**<0.001**						
Calcium (mg/dL)	0.963 (0.884−1.049)	0.391	1.207 (1.038−1.404)	**0.014**	–		–	
Phosphate (mg/dL)	1.146 (1.068−1.230)	**<0.001**	1.026 (0.935−1.125)	0.587	–		–	
ALP (U/L)	1.004 (1.003−1.005)	**<0.001**	1.004 (1.002−1.006)	**<0.001**	1.003 (1.001−1.004)		**<0.001**	
Albumin (g/dL)	1.513 (1.190−1.923)	**0.001**	1.280 (0.945−1.735)	0.111	–		–	
**12 months**								
Calcium (mg/dL)	1.043 (0.935−1.163)	0.452	0.963 (0.845−1.098)	0.573	–		–	
Phosphate (mg/dL)	1.045 (0.968−1.129)	0.257	1.083 (0.985−1.190)	0.101	–		–	
ALP (U/L)	1.000 (0.999−1.000)	0.588	1.000 (0.999−1.000)	0.425	–		–	
Albumin (g/dL)	0.977 (0.744−1.282)	0.865	0.923 (0.657−1.298)	0.647	–		–	

– N = number; PTH: parathyroid hormone; BMI: body mass index; HD: hemodialysis; ALP: alkaline phosphatase. N = 1,689 and N = 1,971 for the initial and final models, respectively. OR – Odds ratio. 95% CI – 95% confidence interval.

## Discussion

SHPT is a common complication of CKD, with a negative impact on morbidity and mortality of patients on dialysis. In this study, we evaluated the role of demographic and clinical factors on the behavior of PTH levels at the beginning and during the first year of HD. In this cohort, almost 20% of incident HD patients had uncontrolled SHPT, which persisted even after 1 year on HD.

Poor adherence to treatment, lack of access to medications, quality of HD treatment and inadequate PTH surveillance are factors that can partially explain the poor CKD-MBD control in this cohort during the first year of HD. Individuals with uncontrolled SHPT were younger, nonwhite, and assisted by the public health system. The limited access of patients with CKD to adequate treatment is critical in our country, which explains the evident contrast regarding SHPT control depending on the paying source of HD treatment. Racial-ethnic differences have already been reported to influence the severity of CKD-MBD. Black people have been shown to develop more severe SHPT^8,9^, although how it reflects on bone turnover status needs to be confirmed^10^. Indeed, a large international cohort with 33,517 patients has revealed that MBD outcomes and PTH trajectories after HD initiation vary according to region and race^6^. Despite the large variation of PTH levels at baseline, a significant decrease in mean levels has been observed in the first year of HD treatment worldwide. In the United States and Europe, PTH levels returned to baseline levels over the course of 4.5 years after HD start, while it remained stable in Japanese patients. Data from Dialysis Outcomes and Practice Patterns Study (DOPPS) has also demonstrated a more stable PTH control in Japanese patients on HD than in patients from other regions^11^.

In this analysis, the patients with PTH >600 pg/mL at 12 months had higher levels of PTH, ALP, phosphate, and albumin when starting HD. Hyperphosphatemia has been described as an important driver to SHPT development. Phosphate retention affects transcriptional and posttranscriptional events, which influence PTH synthesis and stability^12,13^.

Uncontrolled SHPT was more likely (4-fold) in patients with baseline PTH > 600 pg/mL. The development of uncontrolled SHPT at 12 months was 82% lower in those patients with initial PTH below 150 compared to those with an initial PTH between 150 and 600. Unfortunately, due to limited access to health care during conservative care, many patients with CKD in Brazil begin CKD-MBD treatment just after starting HD. Despite the lack of data on medical therapy, it seems that the severity of SHPT impairs the response to drug treatment, reinforcing previous findings^14^. Thus, higher PTH and ALP at baseline were independent factors for uncontrolled SHPT after 1 year of HD, and the longer the exposure to excessive PTH, the greater the systemic consequences, particularly for skeletal health^15^. Our results highlight the importance of focusing on CKD-MBD adequate treatment before starting dialysis during conservative care.

Older age and diabetes were associated with reduced risk of uncontrolled SHPT during the first year of HD in our cohort. As previously described, aging and diabetic nephropathy are factors known to induce adynamic bone disease^16,17^. Oxidative stress and secondary hypogonadism, which are part of aging, stimulate apoptosis of osteoblasts and osteocytes. Diabetes mellitus is also known to enhance osteocyte apoptosis, cause bone fragility and promote changes in the expression of osteocytic genes, including *SOST* ^18^. Moreover, low bone turnover in diabetic patients has been associated with low levels of PTH^19^. Hyperglycemia, insulin deficiency and accumulation of advanced glycation end products induce changes in skeletal micro and macro-architecture, leading to abnormal biomechanical properties and impaired bone strength^20^.

The retrospective and observational design are important limitations of this study, which resulted in missing information, especially about medications used during the first year of HD and dialysate characteristics, such as calcium concentration. However, this is the largest Brazilian ‘real-life’ study assessing the impact of demographic and clinical parameters on PTH behavior in incident HD patients. In Brazil, many patients start HD with already high levels of PTH, suggesting inadequate control of CKD-MBD during the pre-dialysis period. This contributes to the persistence of SHPT after 1 year, in contrast to the better outcomes achieved worldwide. SHPT management is known to be challenging, and a closer monitoring and early initiative to achieve adequate PTH control should be taken since the start of conservative care. Furthermore, inadequate management of medical therapy after initiation of HD is likely to lead to the development of severe SHPT and increased need for PTX in the short term.

## Data Availability

The authors confirm that the data supporting the findings of this study are available within the manuscript.
