# Supplementary material for: Serum parathyroid hormone trajectory during the first year of hemodialysis: a roadmap to severe hyperparathyroidism
Source: J Bras Nefrol. 2025 Jun 13;47(2):e20240182. doi: 10.1590/2175-8239-JBN-2024-0182en (PMC12175965; doi:10.1590/2175-8239-JBN-2024-0182en)
Supplement: Translation

# Translation: Trajetória do paratormônio sérico durante o primeiro ano de hemodiálise: um roteiro para o hiperparatireoidismo grave

**DOI:** 10.1590/2175-8239-JBN-2024-0182pt

**Authors:** Eduardo J. Duque, Maria Eugenia F. Canziani, Ana Beatriz L. Barra, Maria A. Dalboni, Jorge P. Strogoff-de-Matos, Rosilene M. Elias, Rosa M. A. Moysés

**Affiliations:** 1 Universidade de São Paulo, Faculdade de Medicina, Hospital das Clínicas, Laboratório de Fisiopatologia Renal, Departamento de Nefrologia, São Paulo, SP, Brasil.; 2 Universidade Federal de São Paulo, São Paulo, SP, Brasil.; 3 Universidade Federal Fluminense, Rio de Janeiro, RJ, Brasil.; 4 Universidade Nove de Julho, São Paulo, SP, Brasil.

**Keywords:** Hormônio Paratireóideo, Diálise, Hiperparatireoidismo Secundário, Distúrbio Mineral, Ósseo na Doença Renal Crônica

## Abstract

**Histórico:**

Dados sobre os níveis de paratormônio (PTH) no início da hemodiálise (HD) e ao longo do primeiro ano de terapia ainda são escassos. Nossa hipótese é que níveis basais elevados de PTH contribuem para um hiperparatireoidismo mais grave durante esse período.

**Métodos:**

Pacientes incidentes em HD (n = 1.973) foram divididos em 3 grupos de acordo com os valores de PTH (<150, 150–600 e >600 pg/mL).

**Resultados:**

Os níveis de PTH no início do estudo e em um ano foram de 273 (133–508) e 255 (128–471) pg/mL, respectivamente (p = 0,291). Os valores de PTH <150, 150–600 e >600 pg/mL foram encontrados em 28,1, 53,5 e 18,4%, respectivamente, no início do estudo e em 30,7, 52,5 e 16,8% após um ano (p = 0,015). Idade mais jovem, ausência de diabetes, níveis basais elevados de fosfatase alcalina e PTH foram fatores de risco independentes para PTH> 600 pg/mL após um ano de HD.

**ConclusÃO:**

Níveis elevados de PTH no início e após um ano de HD indicam um manejo conservador inadequado antes e durante a diálise, aumentando o risco de necessidade de paratireoidectomia no futuro.

## Introdução

O distúrbio mineral e ósseo na doença renal crônica (DMO-DRC) é um distúrbio sistêmico abrangente que se manifesta em pacientes urêmicos. Essa síndrome compreende deficiência de vitamina D, calcificação vascular, anormalidades na remodelação óssea, metabolismo alterado de cálcio e fosfato e níveis aumentados de fator de crescimento de fibroblastos-23 (FGF-23) e paratormônio (PTH), caracterizando o hiperparatireoidismo secundário (HPTS).

O HPTS danifica diversos sistemas^1^, incluindo a qualidade óssea, o que contribui para o aumento do risco de fraturas em pacientes com DRC. O HPTS resulta em hiperfosfatemia, aumento da calcificação do esqueleto, anemia e pior qualidade de vida. Além disso, foi demonstrado que níveis de PTH >600 pg/mL levam a um aumento de 21% na mortalidade por todas as causas^2^. Estudos revelam que pacientes com HPTS moderado ou grave submetidos à paratireoidectomia (PTX) apresentam aumento da sobrevida, melhora de sintomas como dor óssea e incidência de fraturas^3^, além de melhora na qualidade de vida e na funcionalidade^4,5^.

No Brasil, o grande número de pacientes em diálise aguardando a PTX sugere que o controle do PTH ainda é um desafio. Níveis elevados de PTH em pacientes incidentes em HD estão associados a um controle mais difícil do HPTS a longo prazo^6^, embora dados brasileiros ainda sejam escassos. Nossa hipótese é que os níveis elevados de PTH em pacientes incidentes possam contribuir para a gravidade do HPTS durante o primeiro ano de HD.

## Mêtodos

Este foi um estudo de coorte longitudinal, observacional e retrospectivo de pacientes adultos com doença renal em estágio terminal em HD, acompanhados em 23 unidades de diálise da *Fresenius Medical Care* no Brasil (Rio de Janeiro, São Paulo, Minas Gerais, Bahia, Pernambuco e Distrito Federal). Avaliamos 1.973 dos 4.317 indivíduos adultos que iniciaram a HD entre fevereiro de 2012 e junho de 2017^7^ e completaram um ano de terapia. Durante esse período, houve 661 óbitos (17,4%). Também excluímos da análise pacientes que realizaram diálise peritoneal, foram submetidos a transplante renal, foram transferidos para outras unidades ou não tiveram o PTH medido no início do estudo ou em um ano de acompanhamento. Os dados foram coletados a partir de prontuários eletrônicos e incluíram idade, sexo, raça, diabetes mellitus, índice de massa corporal, fonte de pagamento (Sistema Público de Saúde ou particular), local da primeira diálise (unidade ambulatorial ou hospitalar) e níveis de cálcio, fosfato, albumina, ureia, fosfatase alcalina e PTH.

O paratormônio intacto (PTH; intervalo de referência, RR 15–65 pg/mL) e a 25-vitamina D sérica (RR = 30–60 ng/mL para grupos de risco, inclusive pacientes com DRC) foram medidos por imunoensaio quimioluminescente. Cálcio total sérico (intervalo de referência [RR] = 8,4 – 10,2 mg/dL), fosfatase alcalina sérica (ALP; RR = 35–104 U/L), fosfato sérico (RR = 2,3–4,5 mg/dL) e albumina (RR = 3,4 a 5,4 g/dL) foram medidos por meio de ensaio colorimétrico.

Os pacientes foram divididos em três grupos de acordo com os valores de PTH no início e após 12 meses, utilizando os pontos de corte de <150, 150–600 e >600 pg/mL, que corresponderiam aproximadamente a <2 vezes o limite superior do intervalo de referência (LS), 2–9 vezes o LS e 9 vezes o LS. O HPTS não controlado foi definido como PTH >600 pg/mL.

### Análise Estatística

As variáveis contínuas são expressas como médias e desvios padrão ou como medianas e intervalos interquartis, conforme apropriado. As variáveis categóricas são expressas como valores absolutos e porcentagens, e as diferenças entre os grupos foram verificadas pelo teste Qui-Quadrado. Os valores de PTH em diferentes momentos foram comparados pelo teste de McNemar.

Para avaliar os efeitos das características demográficas e clínicas (variáveis preditivas) nos níveis de PTH, foram utilizadas regressões logísticas univariada e multivariada. No modelo multivariado inicial, todas as variáveis preditivas foram consideradas. Para variáveis preditivas apresentadas em forma numérica e categórica, considerou-se a forma cuja associação com o status do PTH em doze meses foi mais significativa ou com maior tamanho de efeito. Em seguida, as variáveis que não foram significativas foram excluídas uma a uma em ordem de significância (método *backward*). Para todos os testes estatísticos, foi utilizado um nível de significância de 5%. As análises foram realizadas com o SPSS 20.0 e o STATA 17.

## Resultados

Os níveis de PTH no início do estudo e em um ano foram de 273 (133–508) e 255 (128–471) pg/mL, respectivamente (p = 0,291), conforme demonstrado na Figura 1A. Ao iniciar a HD, os níveis de PTH <150, entre 150–600 e >600 pg/mL foram encontrados em 28,1%, 53,5% e 18,4% dos pacientes, respectivamente. Após um ano, esses níveis atingiram 30,7%, 52,5% e 16,8% dos pacientes (p = 0,015). Considerando os pacientes com PTH basal >600 pg/mL, 44,9% e 10,2% alcançaram níveis de 150–600 e <150 pg/mL, respectivamente; 44,9% permaneceram com PTH >600 pg/mL após um ano de acompanhamento (Figura 1B). Em relação aos pacientes com PTH inicial abaixo de 150 pg/mL, 41,6% mudaram para o intervalo de PTH entre 150 e 600 pg/mL, e 2,9% desenvolveram PTH > 600 pg/mL após um ano. Entre os pacientes com PTH inicial entre 150 e 600 pg/mL, 24,7% e 14,5% evoluíram para PTH abaixo de 150 e acima de 600 pg/mL, respectivamente.

**Figura 1:** Níveis de PTH no início e após 1 ano de hemodiálise. A – Os valores de PTH no grupo de estudo permaneceram estáveis após um ano de terapia, sem alterações significativas nos níveis médios em comparação com o valor basal. B – A distribuição dos níveis de PTH aos 12 meses, categorizada por grupos de níveis iniciais de PTH, revelou que, enquanto metade dos pacientes com PTH basal > 600 pg/mL apresentou uma redução em seus níveis séricos, um número equivalente de pacientes de outros grupos aumentou os níveis de PTH acima de 600 pg/mL. Consequentemente, a proporção de pacientes com hiperparatireoidismo não controlado permaneceu igual ao valor basal.

Conforme mostrado na Tabela 1, os pacientes com PTH >600 pg/mL aos 12 meses de HD eram mais jovens, com maior probabilidade de serem não brancos, com menor prevalência de diabetes e fonte de pagamento privada. Além disso, no início do estudo, eles apresentaram PTH, fosfato, ALP e albumina séricos mais elevados. Entretanto, aos 12 meses, não foram observadas diferenças entre os grupos quanto aos níveis séricos de fosfato, ALP ou albumina.

**Tabela 1:** Características demográficas e clínicas por status de pth aos 12 meses

	PTH – 12 meses		Total	p
	≤ 600	> 600		
**Sexo**				0,603
Feminino	652 (39,7)	137 (41,3)	789 (40)	
Masculino	989 (60,3)	195 (58,7)	1.184 (60)	
**Idade (anos)**	57,0 ± 15,4	50,4 ± 15	55,9 ± 15,5	**<0,001**
**Etnia**				**0,024**
Não-branco	913 (55,6)	207 (62,3)	1.120 (56,8)	
Branco	728 (44,4)	125 (37,7)	853 (43,2)	
**IMC (kg/m^2^)**	24,41 ± 4,86	24,85 ± 5,65	24,49 ± 5	0,254
**Diabetes**				**<0,001**
Não	916 (55,8)	245 (73,8)	1.161 (58,8)	
Sim	725 (44,2)	87 (26,2)	812 (41,2)	
**Sistema público**				**<0,001**
Não	623 (38)	88 (26,5)	711 (36)	
Sim	1.018 (62)	244 (73,5)	1.262 (64)	
**1ª HD no hospital**				0,090
Não	465 (29,9)	107 (34,7)	572 (30,7)	
Sim	1.092 (70,1)	201 (65,3)	1.293 (69,3)	
**BASAL**				
**PTH basal (pg/mL)**				**<0,001**
<150	539 (32,8)	16 (4,8)	555 (28,1)	
150 – 600	902 (55)	153 (46,1)	1.055 (53,5)	
>600	200 (12,2)	163 (49,1)	363 (18,4)	
**Cálcio (mg/dL)**	8,82 ± 1,32	8,75 ± 1,37	8,81 ± 1,32	0,819
**Fosfato (mg/dL)**	4,79 ± 1,60	5,15 ± 1,53	4,85 ± 1,59	**<0,001**
**ALP (U/L)**	107,59 ± 76,50	143,33 ± 110,04	113,61 ± 84,14	**<0,001**
**Albumina (g/dL)**	3,57 ± 0,52	3,67 ± 0,50	3,59 ± 0,52	**<0,001**
**12 MESES**				
**Cálcio (mg/dL)**	9,02 ± 1,17	9,08 ± 1,13	9,03 ± 1,16	0,395
**Fosfato (mg/dL)**	4,91 ± 1,54	5,02 ± 1,43	4,93 ± 1,52	0,121
**ALP (U/L)**	180,58 ± 279,45	171,45 ± 266,64	179,03 ± 277,27	0,672
**Albumina (g/dL)**	3,81 ± 0,45	3,81 ± 0,43	3,81 ± 0,45	0,709

Abreviaturas – PTH: paratormônio; IMC: índice de massa corporal; HD: hemodiálise; ALP: fosfatase alcalina. Notas – Os dados são apresentados como média ± DP ou mediana (IIQ), exceto quando indicado de outra forma. Dados em negrito indicam significância.

A análise multivariada (Tabela 2) confirmou que a idade [OR 0,981 (0,973–0,990)] e o diabetes [OR 0,595 (0,445–0,795)] estiveram negativamente associados, enquanto ALP basal [OR 1,003 (1,001–1,004)] e PTH basal > 600 pg/mL [OR 4,003 (3,055–5,325)] estiveram positivamente associados à presença de hiperparatireoidismo não controlado ao final do primeiro ano de terapia.

**Tabela 2:** Análise de regressão logística univariada e multivariada entre parâmetros demográficos/resultados laboratoriais e desenvolvimento de pth > 600 Pg/Ml após 1 ano de diálise

	Análise univariada		Análise multivariada inicial		Análise multivariada final	
	OR (IC 95%)	p	OR (IC 95%)	p	OR (IC 95%)	p
**Masculino (ref. feminino)**	0,938 (0,738–1,193)	0,603	0,955 (0,713–1,280)	0,759	–	–
**Idade (anos)**	0,973 (0,966–0,981)	**<0,001**	0,981 (0,971–0,991)	**<0,001**	0,981 (0,973– 0,990)	**<0,001**
**Branco (ref. não-branco)**	0,757 (0,594–0,965)	**0,025**	0,973 (0,721–1,315)	0,861	–	–
**IMC (kg/m^2^)**	1,017 (0,994–1,041)	0,145	1,025 (0,997–1,053)	0,084	–	–
**Diabetes**	0,449 (0,345–0,584)	**<0,001**	0,581 (0,420–0,803)	**0,001**	0,595 (0,445–0,795)	**<0,001**
**Sistema Público de Saúde**	1,697 (1,304–2,208)	**<0,001**	1,131 (0,819–1,564)	0,454	–	–
**1ª HD no hospital**	0,800 (0,618–1,036)	0,090	0,912 (0,670–1,240)	0,556	–	–
**Basal**						
PTH pg/dL (ref. 151 a 600)		**<0,001**		**<0,001**		**<0,001**
<150	0,175 (0,103–0,296)	**<0,001**	0,185 (0,104–0,329)	**<0,001**	0,181 (0,106–0,307)	**<0,001**
>600	4,805 (3,673–6,285)	**<0,001**	4,037 (2,960–5,507)	**<0,001**	4,033 (3,055–5,325)	**<0,001**
PTH (pg/dL)	1,003 (1,003–1,004)	**<0,001**				
Cálcio (mg/dL)	0,963 (0,884–1,049)	0,391	1,207 (1,038–1,404)	**0,014**	–	–
Fosfato (mg/dL)	1,146 (1,068–1,230)	**<0,001**	1,026 (0,935–1,125)	0,587	–	–
ALP (U/L)	1,004 (1,003–1,005)	**<0,001**	1,004 (1,002–1,006)	**<0,001**	1,003 (1,001–1,004)	**<0,001**
Albumina (g/dL)	1,513 (1,190–1,923)	**0,001**	1,280 (0,945–1,735)	0,111	–	–
**12 meses**						
Cálcio (mg/dL)	1,043 (0,935–1,163)	0,452	0,963 (0,845–1,098)	0,573	–	–
Fosfato (mg/dL)	1,045 (0,968–1,129)	0,257	1,083 (0,985–1,190)	0,101	–	–
ALP (U/L)	1,000 (0,999–1,000)	0,588	1,000 (0,999–1,000)	0,425	–	–
Albumina (g/dL)	0,977 (0,744–1,282)	0,865	0,923 (0,657–1,298)	0,647	–	–

Abreviaturas – N = número; PTH: paratormônio; IMC: índice de massa corporal; HD: hemodiálise; ALP: fosfatase alcalina. N = 1.689 e N = 1.971 para os modelos inicial e final, respectivamente. OR – *Odds Ratio.* IC 95% – Intervalo de confiança de 95%.

## Discussão

O HPTS é uma complicação comum da DRC, com impacto negativo na morbimortalidade dos pacientes em diálise. Neste estudo, avaliamos o papel de fatores demográficos e clínicos no comportamento dos níveis de PTH no início e durante o primeiro ano de HD. Nessa coorte, cerca de 20% dos pacientes incidentes em HD apresentaram HPTS não controlado, que persistiu mesmo após um ano de HD.

A má adesão ao tratamento, a falta de acesso a medicamentos, a qualidade do tratamento de HD e a vigilância inadequada dos níveis de PTH são fatores que podem explicar parcialmente o controle insatisfatório do DMO-DRC nessa coorte durante o primeiro ano de HD. Indivíduos com HPTS não controlado eram mais jovens, não brancos e atendidos pelo sistema público de saúde. O acesso limitado de pacientes com DRC ao tratamento adequado é crítico em nosso país, o que explica o evidente contraste quanto ao controle do HPTS, dependendo da fonte pagadora do tratamento de HD. Já foi relatado que diferenças étnico-raciais influenciam a gravidade do DMO-DRC. Demonstrou-se que pessoas pretas desenvolvem HPTS mais grave^8,9^, embora seja necessário confirmar como isso se reflete no estado de remodelação óssea^10^. De fato, uma grande coorte internacional com 33.517 pacientes revelou que os desfechos do DMO e as trajetórias do PTH após o início da HD variam de acordo com a região e a raça^6^. Apesar da grande variação dos níveis de PTH no início do tratamento, observou-se uma redução significativa nos níveis médios no primeiro ano de tratamento de HD a nível mundial. Nos Estados Unidos e na Europa, os níveis de PTH retornaram aos níveis basais ao longo de 4,5 anos após o início da HD, enquanto permaneceram estáveis nos pacientes japoneses. Dados do *Dialysis Outcomes and Practice Patterns Study* (DOPPS) também demonstraram um controle de PTH mais estável em pacientes japoneses em HD do que em pacientes de outras regiões^11^.

Nesta análise, os pacientes com PTH >600 pg/mL aos 12 meses apresentaram níveis mais elevados de PTH, ALP, fosfato e albumina no início da HD. A hiperfosfatemia foi descrita como um importante impulsionador do desenvolvimento do HPTS. A retenção de fosfato afeta eventos transcricionais e pós-transcricionais, que influenciam a síntese e a estabilidade do PTH^12,13^.

O HPTS não controlado foi mais provável (4 vezes) em pacientes com PTH basal > 600 pg/mL. O desenvolvimento de HPTS não controlado aos 12 meses foi 82% menor nos pacientes com PTH inicial abaixo de 150 em comparação com aqueles com PTH inicial entre 150 e 600. Infelizmente, devido ao acesso limitado aos cuidados de saúde durante o tratamento conservador, muitos pacientes com DRC no Brasil iniciam o tratamento de DMO-DRC somente após o início da HD. Apesar da falta de dados sobre a terapia médica, a gravidade do HPTS parece prejudicar a resposta ao tratamento medicamentoso, reforçando achados anteriores^14^. Assim, níveis mais elevados de PTH e ALP no início do estudo foram fatores independentes para o HPTS não controlado após um ano de HD, e quanto maior a exposição ao PTH excessivo, maiores as consequências sistêmicas, particularmente para a saúde óssea^15^. Nossos resultados destacam a importância de se concentrar no tratamento adequado do DMO-DRC antes de iniciar a diálise, na fase de cuidado conservador.

A idade avançada e o diabetes foram associados a um risco reduzido de HPTS não controlado durante o primeiro ano de HD em nossa coorte. Conforme descrito anteriormente, o envelhecimento e a nefropatia diabética são fatores conhecidos por induzir a doença óssea adinâmica^16,17^. O estresse oxidativo e o hipogonadismo secundário, que fazem parte do envelhecimento, estimulam a apoptose de osteoblastos e osteócitos. Sabe-se também que o diabetes mellitus aumenta a apoptose dos osteócitos, causa fragilidade óssea e promove alterações na expressão de genes osteocíticos, incluindo o *SOST* ^18^. Além disso, a baixa remodelação óssea em pacientes diabéticos tem sido associada a baixos níveis de PTH^19^. A hiperglicemia, a deficiência de insulina e o acúmulo de produtos finais de glicação avançada induzem a alterações na micro e macroarquitetura do esqueleto, levando a propriedades biomecânicas anormais e ao comprometimento da resistência óssea^20^.

O desenho retrospectivo e observacional são limitações importantes deste estudo, resultando na falta de informações, especialmente sobre os medicamentos utilizados durante o primeiro ano de HD e as características do dialisato, como a concentração de cálcio. No entanto, esse é o maior estudo brasileiro de ‘vida real’ que avalia o impacto de parâmetros demográficos e clínicos no comportamento do PTH em pacientes incidentes em HD. No Brasil, muitos pacientes iniciam a HD já com níveis elevados de PTH, o que sugere um controle inadequado do DMO-DRC durante o período pré-diálise. Isso contribui para a persistência do HPTS após um ano, em contraste com os melhores desfechos alcançados em todo o mundo. Sabe-se que o manejo do HPTS é desafiador, e um monitoramento mais rigoroso e uma iniciativa precoce para alcançar o controle adequado do PTH devem ser tomados desde o início do cuidado conservador. Além disso, o manejo inadequado da terapia medicamentosa após o início da HD provavelmente levará ao desenvolvimento de HPTS grave e ao aumento da necessidade de PTX em curto prazo.

## Data Availability

Os autores confirmam que os dados que sustentam os resultados deste estudo estão disponíveis no manuscrito.
